# Tri-μ-oxido-bis­[(5,10,15,20-tetra­phenyl­porphyrinato-κ^4^ *N*)niobium(V)]

**DOI:** 10.1107/S1600536811020538

**Published:** 2011-06-04

**Authors:** Raoudha Soury, Mohamed Salah Belkhiria, Jean-Claude Daran, Habib Nasri

**Affiliations:** a Département de Chimie, Faculté des Sciences de Monastir, Université de Monastir, Avenue de l’Environnement, 5019 Monastir, Tunisia; b Laboratoire de Chimie de Coordination, CNRS UPR 8241, 205 Route de Norbonne, 31077 Toulouse, Cedex 04, France

## Abstract

In the title dinuclear Nb^V^ compound, [Nb_2_(C_44_H_28_N_4_)_2_O_3_], each Nb atom is seven-coordinated with three bridging O atoms and four N atoms from a chelating tetra­phenyl­porphyrinate anion. The Nb—O bond lengths range from 1.757 (6) to 2.331 (6) Å, and the average (niobium–pyrrole N atom) distance is 2.239 Å. In the dinuclear mol­ecule, the Nb⋯Nb separation is 2.8200 (8) Å, and the dihedral angle between the two porphyrinate mean planes is 5.4 (1)°. Weak inter­molecular C—H⋯π inter­actions are present in the crystal structure.

## Related literature

For a review of porphyrin complexes, see: Scheidt (2000 ▶). For the synthesis of niobium(V) porphyrin derivatives, see: Johson & Scheidt (1978 ▶); Lecomte *et al.* (1979 ▶). For comparative bond lengths, see: Allen *et al.* (1987 ▶). For a description of the Cambridge Structural Database, see: Allen (2002 ▶).
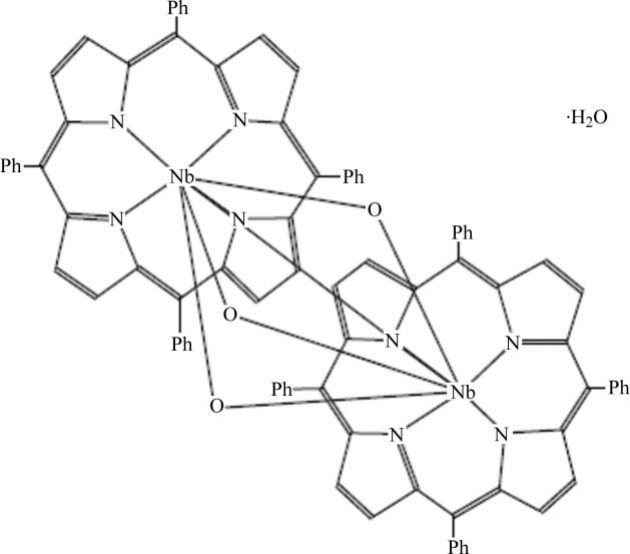


## Experimental

### 

#### Crystal data

[Nb_2_(C_44_H_28_N_4_)_2_O_3_]
*M* *_r_* = 1459.28
Monoclinic, 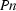

*a* = 14.4823 (9) Å
*b* = 18.4007 (8) Å
*c* = 14.6257 (10) Å
β = 117.823 (7)°
*V* = 3446.9 (4) Å^3^
*Z* = 2
Mo *K*α radiation
μ = 0.39 mm^−1^
*T* = 180 K
0.5 × 0.3 × 0.1 mm

#### Data collection

Bruker APEXII CCD area-detector diffractometer
Absorption correction: multi-scan (*SADABS*; Bruker, 2007 ▶) *T*_min_ = 0.72, *T*_max_ = 1.00
25553 measured reflections
9945 independent reflections
8244 reflections with *I* > 2σ(*I*)
*R*_int_ = 0.055

#### Refinement

*R*[*F* ^2^ > 2σ(*F* ^2^)] = 0.062
*wR*(*F* ^2^) = 0.166
*S* = 1.06
9945 reflections
910 parameters
32 restraints
H-atom parameters constrained
Δρ_max_ = 1.20 e Å^−3^
Δρ_min_ = −0.91 e Å^−3^
Absolute structure: Flack (1983 ▶), 2439 Friedel pairs
Flack parameter: −0.01 (6)

### 

Data collection: *APEX2* (Bruker, 2007 ▶); cell refinement: *SAINT* (Bruker, 2007 ▶); data reduction: *SAINT*; program(s) used to solve structure: *SIR2004* (Burla *et al.*, 2005 ▶); program(s) used to refine structure: *SHELXL97* (Sheldrick, 2008 ▶); molecular graphics: *ORTEPIII* (Burnett & Johnson, 1996 ▶); software used to prepare material for publication: *publCIF* (Westrip, 2010 ▶)..

## Supplementary Material

Crystal structure: contains datablock(s) I, global. DOI: 10.1107/S1600536811020538/xu5216sup1.cif

Structure factors: contains datablock(s) I. DOI: 10.1107/S1600536811020538/xu5216Isup2.hkl

Additional supplementary materials:  crystallographic information; 3D view; checkCIF report

## Figures and Tables

**Table 1:** Selected bond lengths (Å)

Nb1—O1	1.876 (6)
Nb1—O2	1.815 (6)
Nb1—O3	2.331 (6)
Nb1—N1	2.240 (7)
Nb1—N2	2.228 (7)
Nb1—N3	2.261 (7)
Nb1—N4	2.224 (7)
Nb2—O1	2.019 (6)
Nb2—O2	2.182 (6)
Nb2—O3	1.757 (6)
Nb2—N5	2.260 (7)
Nb2—N6	2.226 (7)
Nb2—N7	2.227 (7)
Nb2—N8	2.246 (7)

**Table 2:** Hydrogen-bond geometry (Å, °) *Cg*1 and *Cg*2 are the centroids of the C27-benzene ring and N1-pyrrole ring, respectively.

*D*—H⋯*A*	*D*—H	H⋯*A*	*D*⋯*A*	*D*—H⋯*A*
C22—H22⋯*Cg*1^i^	0.93	2.85	3.752 (14)	164
C40—H40⋯*Cg*2^i^	0.93	2.87	3.681 (14)	147

Symmetry code: (i) 
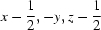
.

**Fig. 1.**
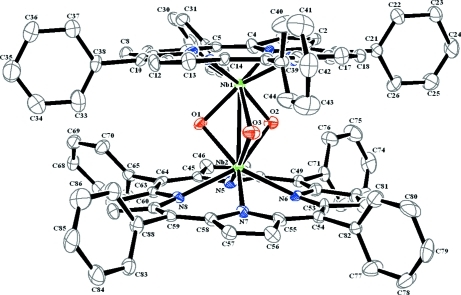
A view of the structure of complex [Nb2O3(C44H28N4)2] showing the atom numbering scheme. Displacement ellipsoids are drawn at 30%. The H atoms have been omitted for clarity.

**Fig. 2.**
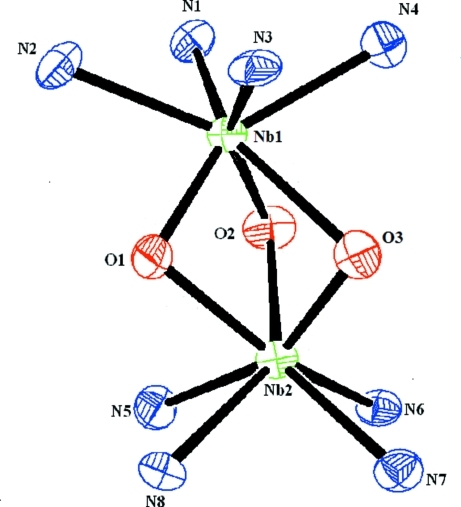
A drawing showing the coordination polyhedrons of the the two niobium atoms Nb1 and Nb2. Displacement ellipsoids are drawn at 50%.

## supplementary crystallographic information

### Comment

The search of the November 2010 release of the Cambridge Structural Database (Allen, 2002) reveals the presence of only one room temperature crystal structure of the [Nb_2_O_3_(C_44_H_28_N_4_)_2_] complex with four 1,2-dichloroethane solvate molecules (Lecomte *et al.*, 1979) with *R* = 0.076 (space group *Pccn*). One year earlier, the room temperature crystal structure of this species was published (Johson & Scheidt, 1978) with *R* = 0.063 (space group *Cc*).

The current redetermination of this Nb(V) porphyrin species at 180 K provides a slightly lower *R* value (*R* = 0.062 based on 8239 independent observed reflections) than the two reported structures.

The asymmetric unit of the structure of (I) contains one complex [Nb_2_O_3_(C_44_H_28_N_4_)_2_]. The average equatorial niobium–pyrrole N atom (Nb—N_p_) distance in (I) is 2.239 (7) Å and each niobium is displaced by 1.01 Å from the 24 atome of the porphyrinato core.

As is clearly seen in Fig. 1 and Fig. 2, the two niobium(V) porphyrin moieties are joined together by three bridging oxo ligand. Thus, each Nb^V^ ion is seven-coordinated. Only one of the bridging oxo ligands forms a nearly symmetric bridge; the other two bridges are quite asymmetric. Unique Nb—O bond lenghts are listed in Table 1. The distance between the two niobium(V) atoms is 2.8200 (8) Å. The two porphyrinato planes are not quite parallel; the dihedral angle between them is 5.4 (1)°. The two porphyrinato rings have a "slipped" configuration with respect to each other; the angles between the normals to the ring passing through the closest niobium atom and the Nb—Nb vector are 16° and 20°.

### Experimental

A solution of NbCl_5_ (1.50 g, 5.55 mmol) in benzonitrile (21 ml) was introduced under argon in a reactor. A solution of the tetraphenylporphyrin (TPP) (1.00 g, 1.62 mmol) in the same solvent (30 ml) was then added. The mixture was heated under reflux for four hours and then hydrolysed (2 ml of water). The resulting solid was chromatographed and recrystallized.

### Refinement

H atoms were placed using assumed geometry with C—H = 0.93 Å. Displacement parameters of the H atoms were set to 1.2 times the isotropic equivalent for the bonded carbon atoms.

### Crystal data

**Table:**

[Nb_2_(C_44_H_28_N_4_)_2_O_3_]	*F*(000) = 1492
*M**_r_* = 1459.28	*D*_x_ = 1.406 Mg m^−^^3^
Monoclinic, *P**n*	Mo *K*α radiation, λ = 0.71073 Å
Hall symbol: P -2yac	Cell parameters from 12579 reflections
*a* = 14.4823 (9) Å	θ = 3.0–32.1°
*b* = 18.4007 (8) Å	µ = 0.39 mm^−^^1^
*c* = 14.6257 (10) Å	*T* = 180 K
β = 117.823 (7)°	Plate, dark purple
*V* = 3446.9 (4) Å^3^	0.5 × 0.3 × 0.1 mm
*Z* = 2	

### Data collection

**Table:**

Bruker APEXII CCD area-detector diffractometer	9945 independent reflections
Radiation source: fine-focus sealed tube	8244 reflections with *I* > 2σ(*I*)
graphite	*R*_int_ = 0.055
Detector resolution: 8.2632 pixels mm^-1^	θ_max_ = 26.0°, θ_min_ = 3.0°
ω scans	*h* = −16→17
Absorption correction: multi-scan (*SADABS*; Bruker, 2007)	*k* = −22→22
*T*_min_ = 0.72, *T*_max_ = 1.00	*l* = −18→15
25553 measured reflections	

### Refinement

**Table:**

Refinement on *F*^2^	Secondary atom site location: difference Fourier map
Least-squares matrix: full	Hydrogen site location: inferred from neighbouring sites
*R*[*F*^2^ > 2σ(*F*^2^)] = 0.062	H-atom parameters constrained
*wR*(*F*^2^) = 0.166	*w* = 1/[σ^2^(*F*_o_^2^) + (0.0709*P*)^2^ + 14.5546*P*] where*P* = (*F*_o_^2^ + 2*F*_c_^2^)/3
*S* = 1.06	(Δ/σ)_max_ < 0.001
9945 reflections	Δρ_max_ = 1.20 e Å^−^^3^
910 parameters	Δρ_min_ = −0.91 e Å^−^^3^
32 restraints	Absolute structure: Flack (1983), 2439 Friedel pairs
Primary atom site location: structure-invariant direct methods	Flack parameter: −0.01 (6)

### Special details

**Table:**

Geometry. All s.u.'s (except the s.u. in the dihedral angle between two l.s. planes) are estimated using the full covariance matrix. The cell s.u.'s are taken into account individually in the estimation of s.u.'s in distances, angles and torsion angles; correlations between s.u.'s in cell parameters are only used when they are defined by crystal symmetry. An approximate (isotropic) treatment of cell s.u.'s is used for estimating s.u.'s involving l.s. planes.
Refinement. Refinement of *F*^2^ against ALL reflections. The weighted *R*-factor *wR* and goodness of fit *S* are based on *F*^2^, conventional *R*-factors *R* are based on *F*, with *F* set to zero for negative *F*^2^. The threshold expression of *F*^2^ > 2σ(*F*^2^) is used only for calculating *R*-factors(gt) *etc*. and is not relevant to the choice of reflections for refinement. *R*-factors based on *F*^2^ are statistically about twice as large as those based on *F*, and *R*- factors based on ALL data will be even larger.

### Fractional atomic coordinates and isotropic or equivalent isotropic displacement parameters (Å^2^)

**Table:**

	*x*	*y*	*z*	*U*_iso_*/*U*_eq_	
Nb1	0.30292 (4)	0.17400 (4)	0.75581 (4)	0.0230 (2)	
Nb2	0.25738 (4)	0.32308 (4)	0.71453 (4)	0.02206 (19)	
O1	0.3090 (5)	0.2549 (3)	0.8366 (5)	0.0382 (16)	
O2	0.3490 (4)	0.2377 (3)	0.6913 (5)	0.0333 (14)	
O3	0.1663 (5)	0.2527 (3)	0.6570 (5)	0.0438 (17)	
N1	0.4324 (5)	0.1081 (4)	0.7547 (6)	0.0287 (16)	
N2	0.3852 (6)	0.1297 (4)	0.9160 (6)	0.0338 (18)	
N3	0.1636 (6)	0.1342 (3)	0.7721 (6)	0.0288 (16)	
N4	0.2133 (5)	0.1139 (4)	0.6083 (6)	0.0284 (16)	
N5	0.4245 (6)	0.3621 (4)	0.7989 (6)	0.0318 (17)	
N6	0.2825 (5)	0.3623 (3)	0.5839 (5)	0.0268 (15)	
N7	0.1144 (6)	0.3904 (4)	0.6303 (6)	0.0298 (16)	
N8	0.2545 (5)	0.3907 (3)	0.8410 (5)	0.0256 (15)	
C1	0.4400 (8)	0.0919 (5)	0.6671 (8)	0.033 (2)	
C2	0.5435 (8)	0.0736 (5)	0.6927 (8)	0.036 (2)	
H2	0.5677	0.0602	0.6464	0.043*	
C3	0.6003 (7)	0.0789 (5)	0.7943 (8)	0.038 (2)	
H3	0.6713	0.0690	0.8318	0.046*	
C4	0.5332 (8)	0.1024 (6)	0.8368 (8)	0.037 (2)	
C5	0.5645 (8)	0.1139 (4)	0.9402 (8)	0.032 (2)	
C6	0.4935 (7)	0.1256 (5)	0.9758 (8)	0.034 (2)	
C7	0.5203 (9)	0.1409 (5)	1.0831 (8)	0.041 (2)	
H7	0.5871	0.1450	1.1384	0.049*	
C8	0.4293 (7)	0.1479 (5)	1.0865 (7)	0.039 (2)	
H8	0.4235	0.1545	1.1466	0.047*	
C9	0.3446 (9)	0.1438 (5)	0.9868 (8)	0.035 (2)	
C10	0.2392 (8)	0.1516 (5)	0.9573 (8)	0.037 (2)	
C11	0.1580 (8)	0.1455 (5)	0.8607 (8)	0.030 (2)	
C12	0.0482 (8)	0.1504 (5)	0.8352 (8)	0.039 (2)	
H12	0.0225	0.1598	0.8816	0.046*	
C13	−0.0082 (8)	0.1394 (5)	0.7355 (8)	0.042 (2)	
H13	−0.0807	0.1387	0.6986	0.051*	
C14	0.0649 (8)	0.1284 (5)	0.6939 (8)	0.031 (2)	
C15	0.0350 (7)	0.1188 (4)	0.5892 (7)	0.0287 (19)	
C16	0.1052 (7)	0.1089 (5)	0.5503 (7)	0.0298 (19)	
C17	0.0775 (7)	0.0961 (5)	0.4450 (8)	0.040 (2)	
H17	0.0104	0.0911	0.3907	0.048*	
C18	0.1665 (7)	0.0926 (5)	0.4390 (7)	0.035 (2)	
H18	0.1715	0.0850	0.3785	0.042*	
C19	0.2527 (8)	0.1023 (6)	0.5388 (8)	0.033 (2)	
C20	0.3556 (8)	0.0931 (5)	0.5668 (8)	0.033 (2)	
C21	0.3834 (7)	0.0793 (5)	0.4818 (8)	0.035 (2)	
C22	0.3753 (9)	0.0110 (6)	0.4399 (9)	0.050 (3)	
H22	0.3515	−0.0272	0.4650	0.060*	
C23	0.4010 (9)	−0.0024 (6)	0.3624 (9)	0.053 (3)	
H23	0.3947	−0.0489	0.3353	0.063*	
C24	0.4372 (10)	0.0554 (7)	0.3244 (10)	0.066 (3)	
H24	0.4545	0.0473	0.2715	0.079*	
C25	0.4468 (12)	0.1241 (7)	0.3661 (12)	0.075 (4)	
H25	0.4725	0.1620	0.3424	0.090*	
C26	0.4191 (10)	0.1369 (7)	0.4416 (9)	0.059 (3)	
H26	0.4235	0.1837	0.4671	0.070*	
C27	0.7459 (9)	0.1612 (5)	0.9975 (9)	0.046 (3)	
H27	0.7206	0.1920	0.9405	0.056*	
C28	0.8498 (9)	0.1609 (6)	1.0673 (9)	0.052 (3)	
H28	0.8939	0.1930	1.0573	0.062*	
C29	0.8929 (11)	0.1146 (8)	1.1530 (10)	0.056 (4)	
H29	0.9643	0.1133	1.1974	0.067*	
C30	0.8228 (9)	0.0705 (6)	1.1684 (9)	0.049 (3)	
H30	0.8475	0.0409	1.2267	0.059*	
C31	0.7198 (8)	0.0698 (5)	1.1004 (7)	0.042 (2)	
H31	0.6761	0.0387	1.1125	0.051*	
C32	0.6773 (7)	0.1135 (5)	1.0136 (7)	0.034 (2)	
C33	0.2005 (12)	0.2385 (8)	1.0674 (11)	0.078 (4)	
H33	0.2107	0.2767	1.0315	0.094*	
C34	0.1731 (12)	0.2528 (8)	1.1436 (12)	0.075 (4)	
H34	0.1643	0.3009	1.1577	0.090*	
C35	0.1584 (12)	0.1991 (8)	1.1994 (11)	0.076 (4)	
H35	0.1441	0.2095	1.2537	0.092*	
C36	0.1656 (12)	0.1274 (8)	1.1715 (11)	0.073 (4)	
H36	0.1527	0.0894	1.2059	0.088*	
C37	0.1910 (10)	0.1122 (7)	1.0953 (10)	0.060 (3)	
H37	0.1940	0.0641	1.0771	0.072*	
C38	0.2128 (8)	0.1685 (6)	1.0439 (8)	0.040 (2)	
C39	−0.0765 (7)	0.1179 (5)	0.5115 (7)	0.032 (2)	
C40	−0.1353 (9)	0.0562 (7)	0.4896 (11)	0.070 (4)	
H40	−0.1046	0.0136	0.5248	0.084*	
C41	−0.2426 (11)	0.0554 (8)	0.4144 (12)	0.080 (5)	
H41	−0.2801	0.0122	0.3981	0.096*	
C42	−0.2883 (9)	0.1160 (8)	0.3683 (10)	0.056 (3)	
H42	−0.3598	0.1166	0.3241	0.067*	
C43	−0.2294 (10)	0.1801 (7)	0.3856 (12)	0.076 (4)	
H43	−0.2580	0.2216	0.3459	0.091*	
C44	−0.1285 (10)	0.1794 (6)	0.4627 (11)	0.065 (4)	
H44	−0.0934	0.2235	0.4832	0.078*	
C45	0.4836 (7)	0.3637 (4)	0.9034 (7)	0.030 (2)	
C46	0.5922 (7)	0.3488 (5)	0.9348 (8)	0.040 (2)	
H46	0.6461	0.3439	1.0019	0.048*	
C47	0.5990 (8)	0.3433 (5)	0.8460 (8)	0.043 (2)	
H47	0.6601	0.3365	0.8410	0.052*	
C48	0.4984 (8)	0.3497 (5)	0.7625 (8)	0.034 (2)	
C49	0.4725 (7)	0.3466 (5)	0.6575 (8)	0.0304 (19)	
C50	0.3732 (8)	0.3522 (6)	0.5766 (8)	0.037 (2)	
C51	0.3462 (8)	0.3518 (5)	0.4704 (8)	0.037 (2)	
H51	0.3933	0.3464	0.4443	0.045*	
C52	0.2435 (7)	0.3604 (5)	0.4133 (8)	0.038 (2)	
H52	0.2063	0.3607	0.3415	0.045*	
C53	0.2009 (8)	0.3690 (6)	0.4855 (8)	0.034 (2)	
C54	0.0953 (8)	0.3809 (5)	0.4539 (7)	0.032 (2)	
C55	0.0559 (8)	0.3913 (5)	0.5241 (7)	0.035 (2)	
C56	−0.0514 (7)	0.4098 (5)	0.4921 (8)	0.040 (2)	
H56	−0.1046	0.4162	0.4250	0.047*	
C57	−0.0576 (7)	0.4156 (5)	0.5804 (7)	0.034 (2)	
H57	−0.1185	0.4249	0.5849	0.041*	
C58	0.0421 (7)	0.4054 (5)	0.6655 (8)	0.030 (2)	
C59	0.0659 (7)	0.4104 (4)	0.7676 (7)	0.0293 (19)	
C60	0.1670 (7)	0.4084 (5)	0.8505 (8)	0.0294 (19)	
C61	0.1965 (7)	0.4265 (5)	0.9552 (8)	0.037 (2)	
H61	0.1513	0.4415	0.9804	0.044*	
C62	0.2997 (7)	0.4184 (5)	1.0113 (7)	0.036 (2)	
H62	0.3392	0.4266	1.0818	0.044*	
C63	0.3394 (7)	0.3935 (5)	0.9391 (7)	0.026 (2)	
C64	0.4404 (7)	0.3792 (4)	0.9720 (7)	0.032 (2)	
C65	0.5182 (7)	0.3802 (5)	1.0863 (7)	0.035 (2)	
C66	0.6035 (7)	0.4271 (5)	1.1273 (8)	0.039 (2)	
H66	0.6148	0.4592	1.0843	0.047*	
C67	0.6702 (8)	0.4258 (6)	1.2302 (8)	0.045 (2)	
H67	0.7265	0.4576	1.2567	0.054*	
C68	0.6574 (9)	0.3788 (7)	1.2968 (9)	0.044 (3)	
H68	0.7050	0.3779	1.3668	0.053*	
C69	0.5731 (9)	0.3337 (6)	1.2573 (9)	0.048 (3)	
H69	0.5614	0.3033	1.3016	0.057*	
C70	0.5047 (9)	0.3323 (6)	1.1529 (8)	0.047 (3)	
H70	0.4497	0.2993	1.1270	0.056*	
C71	0.5589 (7)	0.3314 (5)	0.6317 (8)	0.037 (2)	
C72	0.5948 (9)	0.3860 (7)	0.5932 (10)	0.057 (3)	
H72	0.5658	0.4322	0.5839	0.068*	
C73	0.6738 (10)	0.3726 (8)	0.5680 (10)	0.064 (3)	
H73	0.6962	0.4098	0.5401	0.076*	
C74	0.7191 (10)	0.3070 (8)	0.5828 (11)	0.071 (4)	
H74	0.7740	0.2995	0.5677	0.085*	
C75	0.6841 (11)	0.2509 (7)	0.6205 (12)	0.069 (4)	
H75	0.7128	0.2047	0.6281	0.083*	
C76	0.6055 (10)	0.2640 (6)	0.6471 (11)	0.060 (3)	
H76	0.5839	0.2268	0.6758	0.071*	
C77	0.0062 (10)	0.4507 (6)	0.2897 (9)	0.063 (4)	
H77	0.0467	0.4910	0.3231	0.075*	
C78	−0.0669 (10)	0.4561 (6)	0.1884 (9)	0.064 (4)	
H78	−0.0771	0.5007	0.1550	0.077*	
C79	−0.1257 (11)	0.3975 (8)	0.1344 (9)	0.064 (4)	
H79	−0.1742	0.4010	0.0649	0.077*	
C80	−0.1097 (12)	0.3328 (6)	0.1883 (10)	0.078 (5)	
H80	−0.1485	0.2919	0.1547	0.094*	
C81	−0.0390 (10)	0.3284 (6)	0.2880 (9)	0.057 (3)	
H81	−0.0302	0.2840	0.3216	0.068*	
C82	0.0212 (7)	0.3863 (5)	0.3433 (7)	0.0291 (19)	
C83	−0.0641 (8)	0.4936 (6)	0.7802 (8)	0.044 (2)	
H83	−0.0416	0.5310	0.7526	0.052*	
C84	−0.1427 (9)	0.5056 (7)	0.8061 (9)	0.059 (3)	
H84	−0.1733	0.5513	0.7950	0.071*	
C85	−0.1773 (10)	0.4514 (8)	0.8482 (10)	0.066 (3)	
H85	−0.2305	0.4602	0.8653	0.079*	
C86	−0.1322 (10)	0.3859 (8)	0.8636 (13)	0.072 (4)	
H86	−0.1545	0.3489	0.8920	0.086*	
C87	−0.0532 (9)	0.3720 (6)	0.8380 (10)	0.054 (3)	
H87	−0.0230	0.3261	0.8495	0.064*	
C88	−0.0189 (7)	0.4263 (6)	0.7953 (8)	0.039 (2)	

### Atomic displacement parameters (Å^2^)

**Table:**

	*U*^11^	*U*^22^	*U*^33^	*U*^12^	*U*^13^	*U*^23^
Nb1	0.0245 (4)	0.0207 (4)	0.0247 (4)	0.0001 (3)	0.0123 (3)	0.0003 (3)
Nb2	0.0212 (4)	0.0230 (4)	0.0270 (4)	0.0004 (3)	0.0155 (3)	0.0011 (3)
O1	0.055 (4)	0.028 (3)	0.022 (3)	−0.006 (3)	0.011 (3)	0.003 (2)
O2	0.029 (3)	0.032 (3)	0.047 (4)	−0.002 (2)	0.025 (3)	0.003 (3)
O3	0.041 (4)	0.033 (3)	0.041 (4)	−0.003 (3)	0.005 (3)	−0.001 (3)
N1	0.030 (4)	0.031 (4)	0.031 (4)	0.001 (3)	0.019 (3)	0.008 (3)
N2	0.029 (4)	0.034 (4)	0.033 (4)	0.005 (3)	0.010 (3)	0.014 (3)
N3	0.042 (4)	0.019 (3)	0.034 (4)	−0.003 (3)	0.024 (3)	0.006 (3)
N4	0.024 (4)	0.032 (4)	0.033 (4)	0.007 (3)	0.016 (3)	−0.007 (3)
N5	0.027 (4)	0.032 (4)	0.032 (4)	−0.007 (3)	0.010 (3)	0.001 (3)
N6	0.033 (4)	0.021 (3)	0.031 (4)	−0.004 (3)	0.019 (3)	0.003 (3)
N7	0.032 (4)	0.028 (3)	0.034 (4)	0.007 (3)	0.019 (3)	−0.002 (3)
N8	0.027 (4)	0.021 (3)	0.032 (4)	−0.003 (3)	0.016 (3)	−0.005 (3)
C1	0.037 (5)	0.032 (4)	0.033 (5)	0.000 (4)	0.018 (4)	0.001 (4)
C2	0.047 (6)	0.030 (4)	0.041 (6)	0.004 (4)	0.028 (5)	0.002 (4)
C3	0.033 (5)	0.038 (5)	0.045 (6)	0.002 (4)	0.018 (4)	0.004 (4)
C4	0.027 (5)	0.046 (6)	0.037 (6)	0.004 (4)	0.014 (4)	−0.008 (4)
C5	0.032 (5)	0.023 (4)	0.041 (5)	0.008 (4)	0.018 (4)	0.005 (4)
C6	0.031 (5)	0.029 (5)	0.036 (6)	−0.001 (4)	0.012 (4)	0.011 (4)
C7	0.048 (6)	0.043 (5)	0.032 (5)	−0.003 (4)	0.018 (5)	0.006 (4)
C8	0.040 (5)	0.041 (5)	0.033 (5)	−0.002 (4)	0.014 (4)	0.000 (4)
C9	0.044 (6)	0.026 (4)	0.039 (6)	−0.003 (4)	0.024 (5)	0.009 (4)
C10	0.054 (6)	0.031 (4)	0.039 (6)	0.001 (4)	0.032 (5)	0.007 (4)
C11	0.041 (6)	0.021 (4)	0.042 (6)	−0.006 (4)	0.030 (5)	0.001 (4)
C12	0.041 (6)	0.048 (5)	0.043 (6)	−0.006 (5)	0.034 (5)	−0.010 (4)
C13	0.036 (5)	0.049 (6)	0.042 (6)	−0.005 (4)	0.019 (4)	−0.006 (5)
C14	0.038 (5)	0.031 (5)	0.031 (5)	−0.008 (4)	0.021 (4)	−0.002 (4)
C15	0.030 (5)	0.025 (4)	0.035 (5)	0.003 (3)	0.018 (4)	0.001 (3)
C16	0.034 (5)	0.023 (4)	0.031 (5)	0.001 (3)	0.014 (4)	−0.006 (4)
C17	0.027 (5)	0.051 (6)	0.036 (5)	0.004 (4)	0.010 (4)	0.001 (5)
C18	0.043 (5)	0.032 (4)	0.026 (4)	0.002 (4)	0.013 (4)	−0.002 (4)
C19	0.031 (5)	0.036 (5)	0.040 (6)	0.006 (4)	0.024 (4)	−0.002 (4)
C20	0.039 (5)	0.029 (4)	0.035 (5)	−0.006 (4)	0.019 (4)	0.001 (4)
C21	0.032 (5)	0.039 (5)	0.039 (5)	−0.001 (4)	0.021 (4)	−0.002 (4)
C22	0.060 (7)	0.048 (6)	0.050 (6)	0.004 (5)	0.032 (5)	−0.004 (5)
C23	0.066 (7)	0.052 (6)	0.055 (7)	0.006 (5)	0.040 (6)	−0.007 (5)
C24	0.076 (9)	0.084 (9)	0.068 (8)	−0.004 (7)	0.060 (7)	−0.010 (7)
C25	0.107 (11)	0.065 (8)	0.088 (10)	−0.012 (7)	0.075 (9)	0.002 (7)
C26	0.081 (8)	0.057 (7)	0.058 (7)	−0.020 (6)	0.048 (6)	0.003 (6)
C27	0.052 (6)	0.041 (5)	0.047 (6)	0.002 (4)	0.023 (5)	0.001 (4)
C28	0.054 (7)	0.042 (6)	0.059 (7)	−0.010 (5)	0.027 (5)	−0.008 (5)
C29	0.043 (7)	0.068 (8)	0.047 (7)	0.014 (6)	0.012 (6)	−0.016 (6)
C30	0.051 (6)	0.045 (6)	0.044 (6)	0.017 (5)	0.015 (5)	−0.003 (5)
C31	0.046 (6)	0.037 (5)	0.033 (5)	0.008 (4)	0.010 (4)	0.004 (4)
C32	0.032 (5)	0.035 (5)	0.037 (5)	−0.001 (4)	0.018 (4)	−0.015 (4)
C33	0.100 (7)	0.087 (7)	0.075 (7)	−0.001 (6)	0.064 (6)	−0.009 (5)
C34	0.095 (7)	0.066 (6)	0.080 (7)	0.010 (5)	0.054 (5)	−0.005 (5)
C35	0.088 (7)	0.090 (7)	0.062 (6)	−0.007 (6)	0.044 (5)	−0.002 (5)
C36	0.092 (7)	0.084 (7)	0.065 (6)	−0.004 (5)	0.054 (5)	0.006 (5)
C37	0.077 (6)	0.058 (6)	0.063 (6)	0.003 (5)	0.047 (5)	−0.001 (5)
C38	0.039 (5)	0.056 (6)	0.033 (5)	−0.006 (4)	0.023 (4)	0.000 (5)
C39	0.034 (5)	0.026 (4)	0.036 (5)	−0.007 (3)	0.016 (4)	−0.003 (4)
C40	0.050 (7)	0.053 (6)	0.077 (9)	−0.020 (5)	0.004 (6)	0.013 (6)
C41	0.054 (8)	0.073 (9)	0.082 (10)	−0.007 (7)	0.005 (7)	−0.006 (8)
C42	0.031 (6)	0.086 (9)	0.047 (7)	0.002 (6)	0.015 (5)	−0.001 (7)
C43	0.055 (8)	0.064 (8)	0.081 (10)	0.013 (6)	0.007 (7)	0.010 (7)
C44	0.053 (7)	0.039 (6)	0.078 (9)	0.003 (5)	0.010 (6)	−0.002 (6)
C45	0.032 (5)	0.020 (4)	0.030 (5)	−0.007 (4)	0.009 (4)	−0.004 (4)
C46	0.028 (5)	0.045 (5)	0.043 (6)	0.002 (4)	0.013 (4)	−0.007 (4)
C47	0.043 (6)	0.045 (5)	0.043 (6)	−0.007 (4)	0.022 (5)	0.004 (4)
C48	0.049 (6)	0.023 (4)	0.035 (5)	0.000 (4)	0.023 (4)	−0.003 (4)
C49	0.029 (5)	0.031 (4)	0.045 (5)	−0.002 (4)	0.028 (4)	0.001 (4)
C50	0.036 (6)	0.037 (5)	0.035 (6)	0.005 (4)	0.015 (4)	0.004 (4)
C51	0.043 (6)	0.042 (5)	0.040 (6)	−0.001 (4)	0.030 (5)	0.006 (4)
C52	0.038 (5)	0.046 (5)	0.041 (5)	−0.002 (4)	0.029 (4)	0.003 (4)
C53	0.035 (5)	0.046 (6)	0.024 (5)	−0.002 (4)	0.017 (4)	−0.004 (4)
C54	0.039 (5)	0.026 (4)	0.031 (5)	−0.007 (4)	0.016 (4)	−0.003 (4)
C55	0.033 (5)	0.032 (5)	0.033 (5)	0.010 (4)	0.009 (4)	−0.006 (4)
C56	0.027 (5)	0.044 (5)	0.043 (6)	0.002 (4)	0.012 (4)	−0.008 (4)
C57	0.026 (4)	0.035 (4)	0.043 (5)	0.003 (3)	0.018 (4)	−0.001 (4)
C58	0.024 (4)	0.031 (5)	0.039 (5)	0.001 (4)	0.017 (4)	−0.004 (4)
C59	0.026 (5)	0.022 (4)	0.044 (5)	0.005 (3)	0.020 (4)	0.000 (4)
C60	0.029 (5)	0.025 (4)	0.039 (5)	0.000 (4)	0.020 (4)	0.004 (4)
C61	0.034 (5)	0.044 (5)	0.041 (6)	0.002 (4)	0.024 (4)	−0.006 (4)
C62	0.039 (5)	0.038 (5)	0.029 (5)	0.001 (4)	0.013 (4)	−0.001 (4)
C63	0.024 (5)	0.023 (4)	0.032 (5)	0.002 (3)	0.014 (4)	−0.001 (4)
C64	0.035 (5)	0.024 (4)	0.031 (5)	−0.011 (3)	0.009 (4)	0.005 (4)
C65	0.032 (5)	0.031 (4)	0.034 (5)	0.007 (4)	0.010 (4)	−0.001 (4)
C66	0.038 (5)	0.036 (5)	0.041 (5)	0.001 (4)	0.016 (4)	0.000 (4)
C67	0.031 (5)	0.053 (6)	0.043 (6)	0.002 (4)	0.012 (4)	−0.010 (5)
C68	0.041 (6)	0.061 (7)	0.029 (6)	0.004 (5)	0.016 (5)	0.002 (5)
C69	0.053 (6)	0.047 (6)	0.050 (6)	0.012 (5)	0.030 (5)	0.010 (5)
C70	0.050 (6)	0.047 (6)	0.037 (5)	−0.005 (5)	0.014 (5)	0.005 (4)
C71	0.030 (5)	0.051 (6)	0.040 (5)	0.002 (4)	0.024 (4)	−0.004 (4)
C72	0.049 (6)	0.071 (7)	0.069 (8)	0.007 (6)	0.043 (6)	0.009 (6)
C73	0.058 (7)	0.081 (9)	0.074 (9)	−0.003 (6)	0.050 (7)	0.011 (7)
C74	0.058 (8)	0.102 (11)	0.080 (9)	−0.010 (7)	0.055 (7)	−0.015 (8)
C75	0.067 (8)	0.062 (7)	0.097 (10)	0.005 (6)	0.054 (7)	−0.014 (7)
C76	0.075 (8)	0.044 (6)	0.097 (10)	0.003 (5)	0.072 (7)	−0.002 (6)
C77	0.068 (8)	0.042 (6)	0.052 (7)	−0.013 (5)	0.006 (6)	0.005 (5)
C78	0.080 (9)	0.048 (6)	0.047 (7)	−0.010 (6)	0.015 (6)	0.018 (5)
C79	0.063 (8)	0.091 (10)	0.025 (6)	−0.006 (7)	0.009 (5)	0.000 (6)
C80	0.096 (11)	0.043 (6)	0.053 (8)	−0.005 (6)	0.000 (7)	−0.020 (6)
C81	0.072 (8)	0.047 (6)	0.043 (6)	−0.010 (5)	0.021 (6)	0.000 (5)
C82	0.031 (5)	0.028 (4)	0.034 (5)	0.002 (3)	0.020 (4)	0.000 (4)
C83	0.049 (6)	0.048 (5)	0.042 (6)	−0.007 (4)	0.028 (5)	−0.003 (4)
C84	0.054 (7)	0.075 (8)	0.061 (7)	0.024 (6)	0.038 (6)	0.001 (6)
C85	0.060 (7)	0.097 (10)	0.063 (8)	0.007 (7)	0.047 (6)	0.007 (7)
C86	0.059 (8)	0.078 (9)	0.110 (12)	0.003 (7)	0.066 (8)	0.010 (8)
C87	0.062 (7)	0.054 (6)	0.067 (8)	−0.002 (5)	0.047 (6)	0.000 (6)
C88	0.030 (5)	0.054 (6)	0.038 (5)	0.006 (4)	0.020 (4)	0.001 (4)

### Geometric parameters (Å, °)

**Table:**

Nb1—O1	1.876 (6)	C36—C37	1.356 (18)
Nb1—O2	1.815 (6)	C36—H36	0.9300
Nb1—O3	2.331 (6)	C37—C38	1.400 (16)
Nb1—N1	2.240 (7)	C37—H37	0.9300
Nb1—N2	2.228 (7)	C39—C44	1.362 (14)
Nb1—N3	2.261 (7)	C39—C40	1.365 (13)
Nb1—N4	2.224 (7)	C40—C41	1.425 (17)
Nb1—Nb2	2.8200 (8)	C40—H40	0.9300
Nb2—O1	2.019 (6)	C41—C42	1.311 (19)
Nb2—O2	2.182 (6)	C41—H41	0.9300
Nb2—O3	1.757 (6)	C42—C43	1.409 (19)
Nb2—N5	2.260 (7)	C42—H42	0.9300
Nb2—N6	2.226 (7)	C43—C44	1.367 (16)
Nb2—N7	2.227 (7)	C43—H43	0.9300
Nb2—N8	2.246 (7)	C44—H44	0.9300
N1—C1	1.369 (12)	C45—C64	1.438 (15)
N1—C4	1.395 (12)	C45—C46	1.445 (14)
N2—C6	1.396 (12)	C46—C47	1.352 (15)
N2—C9	1.433 (14)	C46—H46	0.9300
N3—C11	1.352 (12)	C47—C48	1.402 (15)
N3—C14	1.355 (12)	C47—H47	0.9300
N4—C16	1.393 (11)	C48—C49	1.401 (14)
N4—C19	1.395 (12)	C49—C50	1.375 (14)
N5—C45	1.359 (11)	C49—C71	1.492 (13)
N5—C48	1.419 (13)	C50—C51	1.413 (15)
N6—C53	1.377 (12)	C51—C52	1.332 (13)
N6—C50	1.378 (13)	C51—H51	0.9300
N7—C55	1.379 (12)	C52—C53	1.460 (14)
N7—C58	1.392 (12)	C52—H52	0.9300
N8—C60	1.376 (11)	C53—C54	1.393 (14)
N8—C63	1.389 (11)	C54—C55	1.401 (14)
C1—C20	1.404 (14)	C54—C82	1.469 (13)
C1—C2	1.406 (14)	C55—C56	1.439 (13)
C2—C3	1.323 (14)	C56—C57	1.340 (14)
C2—H2	0.9300	C56—H56	0.9300
C3—C4	1.444 (15)	C57—C58	1.412 (13)
C3—H3	0.9300	C57—H57	0.9300
C4—C5	1.377 (14)	C58—C59	1.370 (14)
C5—C6	1.369 (15)	C59—C60	1.400 (13)
C5—C32	1.478 (13)	C59—C88	1.489 (13)
C6—C7	1.458 (15)	C60—C61	1.424 (14)
C7—C8	1.348 (15)	C61—C62	1.336 (13)
C7—H7	0.9300	C61—H61	0.9300
C8—C9	1.402 (14)	C62—C63	1.490 (14)
C8—H8	0.9300	C62—H62	0.9300
C9—C10	1.386 (15)	C63—C64	1.334 (13)
C10—C11	1.357 (14)	C64—C65	1.519 (13)
C10—C38	1.516 (14)	C65—C66	1.393 (13)
C11—C12	1.457 (14)	C65—C70	1.394 (15)
C12—C13	1.312 (14)	C66—C67	1.357 (14)
C12—H12	0.9300	C66—H66	0.9300
C13—C14	1.461 (15)	C67—C68	1.379 (16)
C13—H13	0.9300	C67—H67	0.9300
C14—C15	1.395 (13)	C68—C69	1.361 (16)
C15—C16	1.389 (13)	C68—H68	0.9300
C15—C39	1.478 (12)	C69—C70	1.379 (15)
C16—C17	1.418 (14)	C69—H69	0.9300
C17—C18	1.334 (14)	C70—H70	0.9300
C17—H17	0.9300	C71—C72	1.368 (16)
C18—C19	1.421 (13)	C71—C76	1.378 (14)
C18—H18	0.9300	C72—C73	1.379 (16)
C19—C20	1.359 (14)	C72—H72	0.9300
C20—C21	1.496 (14)	C73—C74	1.342 (18)
C21—C22	1.380 (14)	C73—H73	0.9300
C21—C26	1.420 (14)	C74—C75	1.374 (19)
C22—C23	1.370 (15)	C74—H74	0.9300
C22—H22	0.9300	C75—C76	1.385 (17)
C23—C24	1.410 (17)	C75—H75	0.9300
C23—H23	0.9300	C76—H76	0.9300
C24—C25	1.383 (18)	C77—C78	1.364 (16)
C24—H24	0.9300	C77—C82	1.381 (14)
C25—C26	1.360 (18)	C77—H77	0.9300
C25—H25	0.9300	C78—C79	1.374 (17)
C26—H26	0.9300	C78—H78	0.9300
C27—C28	1.368 (16)	C79—C80	1.386 (19)
C27—C32	1.426 (15)	C79—H79	0.9300
C27—H27	0.9300	C80—C81	1.336 (17)
C28—C29	1.398 (18)	C80—H80	0.9300
C28—H28	0.9300	C81—C82	1.376 (13)
C29—C30	1.398 (19)	C81—H81	0.9300
C29—H29	0.9300	C83—C88	1.370 (14)
C30—C31	1.352 (14)	C83—C84	1.375 (15)
C30—H30	0.9300	C83—H83	0.9300
C31—C32	1.381 (13)	C84—C85	1.383 (18)
C31—H31	0.9300	C84—H84	0.9300
C33—C38	1.366 (17)	C85—C86	1.339 (18)
C33—C34	1.37 (2)	C85—H85	0.9300
C33—H33	0.9300	C86—C87	1.384 (17)
C34—C35	1.36 (2)	C86—H86	0.9300
C34—H34	0.9300	C87—C88	1.387 (15)
C35—C36	1.40 (2)	C87—H87	0.9300
C35—H35	0.9300		
			
O2—Nb1—O1	83.9 (3)	C30—C31—H31	118.8
O2—Nb1—N4	90.4 (3)	C32—C31—H31	118.8
O1—Nb1—N4	147.1 (3)	C31—C32—C27	117.6 (9)
O2—Nb1—N2	129.5 (3)	C31—C32—C5	122.6 (9)
O1—Nb1—N2	77.5 (3)	C27—C32—C5	119.8 (9)
N4—Nb1—N2	128.4 (3)	C38—C33—C34	120.4 (14)
O2—Nb1—N1	80.5 (3)	C38—C33—H33	119.8
O1—Nb1—N1	129.8 (3)	C34—C33—H33	119.8
N4—Nb1—N1	80.5 (3)	C35—C34—C33	122.2 (14)
N2—Nb1—N1	76.7 (3)	C35—C34—H34	118.9
O2—Nb1—N3	145.7 (3)	C33—C34—H34	118.9
O1—Nb1—N3	88.4 (3)	C34—C35—C36	117.2 (14)
N4—Nb1—N3	78.2 (3)	C34—C35—H35	121.4
N2—Nb1—N3	80.6 (3)	C36—C35—H35	121.4
N1—Nb1—N3	128.1 (3)	C37—C36—C35	121.4 (13)
O2—Nb1—O3	71.8 (3)	C37—C36—H36	119.3
O1—Nb1—O3	71.0 (2)	C35—C36—H36	119.3
N4—Nb1—O3	76.4 (2)	C36—C37—C38	120.2 (12)
N2—Nb1—O3	139.6 (3)	C36—C37—H37	119.9
N1—Nb1—O3	143.5 (3)	C38—C37—H37	119.9
N3—Nb1—O3	74.1 (3)	C33—C38—C37	118.4 (11)
O2—Nb1—Nb2	50.68 (19)	C33—C38—C10	121.1 (10)
O1—Nb1—Nb2	45.65 (18)	C37—C38—C10	120.2 (10)
N4—Nb1—Nb2	107.68 (18)	C44—C39—C40	115.8 (10)
N2—Nb1—Nb2	122.5 (2)	C44—C39—C15	122.2 (8)
N1—Nb1—Nb2	129.65 (18)	C40—C39—C15	121.9 (9)
N3—Nb1—Nb2	101.91 (17)	C39—C40—C41	121.8 (11)
O3—Nb1—Nb2	38.44 (15)	C39—C40—H40	119.1
O3—Nb2—O1	81.4 (3)	C41—C40—H40	119.1
O3—Nb2—O2	76.7 (3)	C42—C41—C40	119.7 (13)
O1—Nb2—O2	71.9 (3)	C42—C41—H41	120.2
O3—Nb2—N6	102.4 (3)	C40—C41—H41	120.2
O1—Nb2—N6	145.0 (3)	C41—C42—C43	120.1 (11)
O2—Nb2—N6	75.3 (2)	C41—C42—H42	119.9
O3—Nb2—N7	81.3 (3)	C43—C42—H42	119.9
O1—Nb2—N7	134.4 (3)	C44—C43—C42	117.6 (12)
O2—Nb2—N7	142.2 (3)	C44—C43—H43	121.2
N6—Nb2—N7	80.0 (3)	C42—C43—H43	121.2
O3—Nb2—N8	120.3 (3)	C39—C44—C43	123.9 (11)
O1—Nb2—N8	76.0 (3)	C39—C44—H44	118.0
O2—Nb2—N8	140.6 (2)	C43—C44—H44	118.0
N6—Nb2—N8	126.9 (3)	N5—C45—C64	122.6 (9)
N7—Nb2—N8	77.1 (3)	N5—C45—C46	112.0 (9)
O3—Nb2—N5	150.3 (3)	C64—C45—C46	125.4 (8)
O1—Nb2—N5	81.6 (3)	C47—C46—C45	105.4 (9)
O2—Nb2—N5	74.9 (2)	C47—C46—H46	127.3
N6—Nb2—N5	78.6 (3)	C45—C46—H46	127.3
N7—Nb2—N5	127.4 (3)	C46—C47—C48	108.6 (10)
N8—Nb2—N5	78.5 (3)	C46—C47—H47	125.7
O3—Nb2—Nb1	55.5 (2)	C48—C47—H47	125.7
O1—Nb2—Nb1	41.65 (18)	C49—C48—C47	126.1 (10)
O2—Nb2—Nb1	40.07 (16)	C49—C48—N5	123.7 (9)
N6—Nb2—Nb1	112.57 (18)	C47—C48—N5	110.2 (9)
N7—Nb2—Nb1	136.36 (19)	C50—C49—C48	125.3 (10)
N8—Nb2—Nb1	117.08 (18)	C50—C49—C71	117.4 (10)
N5—Nb2—Nb1	96.21 (19)	C48—C49—C71	117.2 (9)
Nb1—O1—Nb2	92.7 (3)	C49—C50—N6	126.5 (10)
Nb1—O2—Nb2	89.3 (3)	C49—C50—C51	125.9 (10)
Nb2—O3—Nb1	86.0 (2)	N6—C50—C51	107.5 (8)
C1—N1—C4	106.3 (8)	C52—C51—C50	110.1 (10)
C1—N1—Nb1	123.9 (6)	C52—C51—H51	124.9
C4—N1—Nb1	125.2 (6)	C50—C51—H51	124.9
C6—N2—C9	106.0 (8)	C51—C52—C53	106.5 (9)
C6—N2—Nb1	124.9 (7)	C51—C52—H52	126.7
C9—N2—Nb1	119.7 (6)	C53—C52—H52	126.7
C11—N3—C14	108.0 (8)	N6—C53—C54	129.4 (9)
C11—N3—Nb1	120.5 (6)	N6—C53—C52	107.4 (9)
C14—N3—Nb1	125.4 (6)	C54—C53—C52	123.1 (9)
C16—N4—C19	105.7 (8)	C53—C54—C55	122.5 (9)
C16—N4—Nb1	127.2 (6)	C53—C54—C82	120.1 (9)
C19—N4—Nb1	122.1 (6)	C55—C54—C82	117.4 (9)
C45—N5—C48	103.7 (8)	N7—C55—C54	125.3 (9)
C45—N5—Nb2	125.0 (6)	N7—C55—C56	111.8 (9)
C48—N5—Nb2	123.2 (6)	C54—C55—C56	122.8 (9)
C53—N6—C50	108.4 (8)	C57—C56—C55	104.7 (9)
C53—N6—Nb2	121.4 (6)	C57—C56—H56	127.7
C50—N6—Nb2	124.5 (6)	C55—C56—H56	127.7
C55—N7—C58	104.0 (8)	C56—C57—C58	109.8 (9)
C55—N7—Nb2	123.6 (6)	C56—C57—H57	125.1
C58—N7—Nb2	124.8 (6)	C58—C57—H57	125.1
C60—N8—C63	107.4 (8)	C59—C58—N7	124.6 (8)
C60—N8—Nb2	125.9 (6)	C59—C58—C57	125.8 (9)
C63—N8—Nb2	121.6 (5)	N7—C58—C57	109.6 (9)
N1—C1—C20	124.3 (9)	C58—C59—C60	124.8 (9)
N1—C1—C2	110.1 (8)	C58—C59—C88	119.3 (8)
C20—C1—C2	125.6 (10)	C60—C59—C88	115.6 (9)
C3—C2—C1	108.0 (9)	N8—C60—C59	124.0 (9)
C3—C2—H2	126.0	N8—C60—C61	109.7 (8)
C1—C2—H2	126.0	C59—C60—C61	126.3 (9)
C2—C3—C4	108.3 (9)	C62—C61—C60	108.7 (9)
C2—C3—H3	125.8	C62—C61—H61	125.6
C4—C3—H3	125.8	C60—C61—H61	125.6
C5—C4—N1	127.6 (9)	C61—C62—C63	106.9 (8)
C5—C4—C3	125.2 (9)	C61—C62—H62	126.5
N1—C4—C3	107.2 (9)	C63—C62—H62	126.5
C6—C5—C4	121.4 (9)	C64—C63—N8	131.1 (9)
C6—C5—C32	119.8 (9)	C64—C63—C62	121.7 (9)
C4—C5—C32	118.8 (9)	N8—C63—C62	107.1 (7)
C5—C6—N2	126.4 (10)	C63—C64—C45	123.2 (9)
C5—C6—C7	124.7 (9)	C63—C64—C65	121.3 (10)
N2—C6—C7	108.7 (9)	C45—C64—C65	115.4 (8)
C8—C7—C6	106.5 (9)	C66—C65—C70	118.5 (9)
C8—C7—H7	126.8	C66—C65—C64	122.3 (9)
C6—C7—H7	126.8	C70—C65—C64	119.2 (8)
C7—C8—C9	110.6 (10)	C67—C66—C65	119.6 (10)
C7—C8—H8	124.7	C67—C66—H66	120.2
C9—C8—H8	124.7	C65—C66—H66	120.2
C10—C9—C8	128.2 (10)	C66—C67—C68	122.4 (10)
C10—C9—N2	123.9 (9)	C66—C67—H67	118.8
C8—C9—N2	107.9 (9)	C68—C67—H67	118.8
C11—C10—C9	127.5 (10)	C69—C68—C67	118.1 (10)
C11—C10—C38	116.9 (9)	C69—C68—H68	120.9
C9—C10—C38	115.6 (9)	C67—C68—H68	120.9
N3—C11—C10	126.9 (10)	C68—C69—C70	121.4 (11)
N3—C11—C12	108.1 (8)	C68—C69—H69	119.3
C10—C11—C12	125.0 (9)	C70—C69—H69	119.3
C13—C12—C11	108.3 (9)	C69—C70—C65	119.9 (10)
C13—C12—H12	125.9	C69—C70—H70	120.0
C11—C12—H12	125.9	C65—C70—H70	120.0
C12—C13—C14	106.7 (9)	C72—C71—C76	118.4 (10)
C12—C13—H13	126.6	C72—C71—C49	119.4 (9)
C14—C13—H13	126.6	C76—C71—C49	122.2 (9)
N3—C14—C15	126.9 (9)	C71—C72—C73	120.1 (12)
N3—C14—C13	108.8 (8)	C71—C72—H72	119.9
C15—C14—C13	124.2 (9)	C73—C72—H72	119.9
C16—C15—C14	123.7 (8)	C74—C73—C72	121.5 (12)
C16—C15—C39	115.4 (8)	C74—C73—H73	119.3
C14—C15—C39	120.9 (9)	C72—C73—H73	119.3
C15—C16—N4	124.9 (8)	C73—C74—C75	119.7 (12)
C15—C16—C17	125.2 (8)	C73—C74—H74	120.1
N4—C16—C17	109.9 (8)	C75—C74—H74	120.1
C18—C17—C16	106.7 (8)	C74—C75—C76	119.2 (12)
C18—C17—H17	126.6	C74—C75—H75	120.4
C16—C17—H17	126.6	C76—C75—H75	120.4
C17—C18—C19	109.7 (9)	C71—C76—C75	121.0 (11)
C17—C18—H18	125.1	C71—C76—H76	119.5
C19—C18—H18	125.1	C75—C76—H76	119.5
C20—C19—N4	124.2 (9)	C78—C77—C82	121.0 (10)
C20—C19—C18	127.4 (10)	C78—C77—H77	119.5
N4—C19—C18	107.9 (8)	C82—C77—H77	119.5
C19—C20—C1	127.9 (10)	C77—C78—C79	121.7 (11)
C19—C20—C21	116.9 (9)	C77—C78—H78	119.1
C1—C20—C21	115.1 (9)	C79—C78—H78	119.1
C22—C21—C26	117.8 (10)	C78—C79—C80	117.0 (10)
C22—C21—C20	121.6 (9)	C78—C79—H79	121.5
C26—C21—C20	120.6 (9)	C80—C79—H79	121.5
C23—C22—C21	122.1 (11)	C81—C80—C79	120.8 (11)
C23—C22—H22	118.9	C81—C80—H80	119.6
C21—C22—H22	118.9	C79—C80—H80	119.6
C22—C23—C24	119.0 (11)	C80—C81—C82	123.1 (11)
C22—C23—H23	120.5	C80—C81—H81	118.4
C24—C23—H23	120.5	C82—C81—H81	118.4
C25—C24—C23	119.7 (11)	C81—C82—C77	116.3 (9)
C25—C24—H24	120.1	C81—C82—C54	122.1 (8)
C23—C24—H24	120.1	C77—C82—C54	121.6 (8)
C26—C25—C24	120.6 (12)	C88—C83—C84	119.7 (10)
C26—C25—H25	119.7	C88—C83—H83	120.1
C24—C25—H25	119.7	C84—C83—H83	120.1
C25—C26—C21	120.7 (12)	C83—C84—C85	121.7 (11)
C25—C26—H26	119.6	C83—C84—H84	119.2
C21—C26—H26	119.6	C85—C84—H84	119.2
C28—C27—C32	118.9 (10)	C86—C85—C84	118.4 (11)
C28—C27—H27	120.6	C86—C85—H85	120.8
C32—C27—H27	120.6	C84—C85—H85	120.8
C27—C28—C29	123.2 (12)	C85—C86—C87	121.3 (13)
C27—C28—H28	118.4	C85—C86—H86	119.3
C29—C28—H28	118.4	C87—C86—H86	119.3
C28—C29—C30	116.4 (11)	C86—C87—C88	120.3 (11)
C28—C29—H29	121.8	C86—C87—H87	119.8
C30—C29—H29	121.8	C88—C87—H87	119.8
C31—C30—C29	121.4 (11)	C83—C88—C87	118.6 (10)
C31—C30—H30	119.3	C83—C88—C59	121.7 (9)
C29—C30—H30	119.3	C87—C88—C59	119.7 (9)
C30—C31—C32	122.5 (11)		

### Hydrogen-bond geometry (Å, °)

**Table:**

Cg1 and Cg2 are the centroids of the C27-benzene ring and N1-pyrrole ring, respectively.

**Table:**

*D*—H···*A*	*D*—H	H···*A*	*D*···*A*	*D*—H···*A*
C22—H22···Cg1^i^	0.93	2.85	3.752 (14)	164
C40—H40···Cg2^i^	0.93	2.87	3.681 (14)	147

Symmetry codes: (i) *x*−1/2, −*y*, *z*−1/2.
